# Persistent Testosterone Suppression After Cessation of Androgen Deprivation Therapy for Prostate Cancer

**DOI:** 10.7759/cureus.32699

**Published:** 2022-12-19

**Authors:** Jessica Delgado, Jesse Ory, Justin Loloi, Nicholas A Deebel, Ari Bernstein, Sirpi Nackeeran, Isaac Zucker, Ranjith Ramasamy

**Affiliations:** 1 Urology, University of Miami, Miami, USA; 2 Urology, Dalhousie University, Halifax, CAN; 3 Urology, Montefiore Medical Center, New York, USA; 4 Urology, Atrium Health Wake Forest Baptist, Winston-Salem, USA; 5 Urology, New York University (NYU) Langone, New York, USA; 6 Medical Education, University of Miami Miller School of Medicine, Miami, USA; 7 Medicine, Florida International University, Miami, USA

**Keywords:** prostate cancer, hormonal, antineoplastic agents, testosterone, hypogonadism

## Abstract

Introduction

Many men receiving temporary androgen deprivation therapy (ADT) for localized prostate cancer fail to achieve baseline testosterone levels after cessation. Testosterone recovery in men with localized prostate cancer receiving temporary ADT was assessed.

Methods

A global federated health research network (TriNetX) was used to identify men diagnosed with prostate cancer undergoing temporary ADT. Two cohorts were identified: men receiving luteinizing hormone-releasing hormone (LHRH) antagonists or LHRH agonists, and men receiving combined ADT (LHRH agonist and antiandrogens). Further stratification was based on a treatment duration of six months (short-term) or 18 months (long-term) to compare testosterone (T) recovery profiles five years after ADT cessation.

Results

A total of 28,583 men received LHRH agonist or antagonist therapy alone, and 20,188 men received combination ADT. A total of 46.7% of men who received short-term LHRH agonists or antagonists and 40.6% of men who received short-term combined ADT, recovered to mean baseline T levels at five years. Only men who received short-term LHRH agonists/antagonists recovered to eugonadal levels at the five-year follow-up. Around 50% of men who received long-term LHRH agonist/antagonist therapy and 10.7% of men who received combined ADT, recovered to mean baseline T levels at five years. However, neither group recovered to eugonadal T levels.

Conclusions

At the five-year follow-up after ADT cessation, most patients failed to recover to their mean baseline and eugonadal T levels. Given that testosterone deficiency is associated with metabolically adverse changes in body composition, increased insulin resistance, impaired bone health, and hypogonadal symptoms, serum T levels must be closely monitored in men receiving ADT following treatment cessation.

## Introduction

Prostate cancer (PCa) is the second most common cancer in men following non-melanoma skin cancer [1]. A total of 11.6% of males will be affected in their lifetime, a rate expected to increase with the aging population [1,2]. In its early stages, localized prostate cancer is a hormone-sensitive disease. While definitive therapies such as radical prostatectomy (RP) or radiation therapy (RT) can result in excellent disease-free survival, androgen deprivation therapy (ADT) is often utilized adjunctively in select circumstances to improve outcomes in men with localized PCa. Specifically, ADT is typically given alongside RT for intermediate to high-risk prostate cancer to radiosensitize prostate tissue thereby improving the efficacy of RT [3-6]. Using ADT in this setting is meant to be temporary, being employed anywhere from six to 18 months [5]. Mechanistically, ADT regimens such as those including luteinizing hormone-releasing hormone analogs typically act to imitate hormones secreted by the hypothalamus thus inhibiting the secretion of testosterone from the testicles, resulting in androgen suppression [7].

Recent literature has suggested that testosterone recovery after the temporary use of ADT is not guaranteed. A significant proportion of men fail to return to their baseline testosterone levels years after ADT cessation [2,8]. These men, if properly identified, could benefit from testosterone therapy if symptomatic, as this is oncologically safe in hypogonadal men with previously treated localized prostate cancer or on active surveillance [9]. We currently do not know which men will suffer from persistent hypogonadism after temporary ADT [2]. Whether or not the type or duration of ADT influences testosterone recovery is poorly understood.

This study aimed to investigate testosterone recovery in men with prostate cancer following various temporary ADT modalities and treatment durations. We hypothesized that the majority of patients treated with long-term ADT would have incomplete T recovery following ADT cessation.

## Materials and methods

We accessed data from the TriNetX Analytics Network, a global federated database that captures anonymized data from electronic medical records (EMR) amongt 54 healthcare organizations (HCOs) in the USA totaling 74 million patients. The TriNetX data includes diagnoses (using International Classification of Diseases, 10th Edition, Clinical Modification (ICD-10-CM) codes), demographics, procedures, medications, and measurements. The HCOs include several hospitals, primary care, and specialist providers who contribute data from patients regardless of insurance status. To comply with legal frameworks and ethical guidelines guarding against data re-identification, the identity of participating HCOs and their contribution to each dataset is not disclosed.

We initially evaluated three separate cohorts for comparison amongst men with a diagnosis of prostate cancer who received ADT. These three groups included: 1) combined ADT (antiandrogen plus an LHRH agonist), 2) LHRH agonist alone, and 3) LHRH antagonist alone. The following antiandrogens were included: flutamide, bicalutamide, or nilutamide. The LHRH agonists included leuprolide, goserelin, histrelin, or triptorelin. the LHRH antagonists included degarelix and relugolix. Each ADT group was separated by the duration of time on ADT (six or 18 months). Required use for either six or 18 months was the inclusionary criteria for all hormone therapies including antiandrogens. Due to the limited number of men on LHRH antagonists alone, we combined these men with those on LHRH agonist therapy alone for a total of two ADT groups and two-time frames of treatment (Table 1).

**Table 1:** Patient demographics and comorbidities by ADT cohort HTN: Hypertension, CAD: Coronary artery disease, MDD: Major depressive disorder, SD: Standard deviation, ADT: Androgen deprivation therapy, LHRH: Luteinizing hormone-releasing hormone All values that fall between 1 to 10 patients are censored and rounded up to 10 in TriNetX, and therefore have been represented here as <10.

Characteristics	LHRH Antagonists 6 months	LHRH Antagonists 18 months	LHRH Agonists 6 months	LHRH Agonists 18 months	Combined ADT 6 months	Combined ADT 18 months
Number of patients (N)	N=1,297	N=40	N=23,394	N=3,852	N=16,585	N=3,603
Age at index, mean (SD)	70.8 + 9.23	73.0 + 9.08	71.2 + 9.19	72.5 + 9.04	71.3 ±9.26	72.2 ±8.92
Race	-	-	-	-	-	-
White	643 (50%)	22 (5%)	15,005 (66%)	2,614 (69%)	10,607 (65%)	2,310 (65%)
Unknown Race	500 (39%)	16 (40%)	3,720 (16%)	474 (12%)	2,229 (14%)	429 (12%)
Black or African American	141 (11%)	<10 (<25%)	3,489 (15%)	640 (17%)	3,137 (19%)	762 (21%)
Ethnicity	-	-	-	-	-	-
Not Hispanic or Latino	769 (59%)	17 (43%)	16,683 (74%)	2,919 (77%)	11,970 (73%)	2,736 (76%)
Hispanic or Latino	31 (2%)	<10 (<25%)	988 (4%)	193 (5%)	705 (4%)	169 (5%)
Comorbidities	-	-	-	-	-	-
HTN	524 (40%)	18 (45%)	10,082 (45%)	2,261 (60%)	8,054 (49%)	2,144 (60%)
Diabetes	201 (15%)	<10 (<25%)	4,023 (18%)	915 (24%)	3,106 (19%)	866 (24%)
Hyperlipidemia	346 (27%)	14 (35%)	6,971 (31%)	1,583 (42%)	5,398 (33%)	1,452 (41%)
Sleep Apnea	118 (9%)	<10 (<25%)	2,187 (10%)	833 (22%)	1,583 (10%)	431 (12%)
CAD	204 (16%)	<10 (<25%)	3,735 (7%)	833 (2%)	2,975 (18%)	795 (22%)
Stroke	36 (3%)	0	767 (3%)	197 (5%)	673 (4%)	184 (5%)
MDD	16 (1%)	<10 (<25%)	246 (1%)	79 (2%)	222 (1%)	65 (2%)
Anxiety Disorder	90 (7%)	<10 (<25%)	1,659 (7%)	464 (12%)	1,264 (8%)	387 (11%)
Overweight/Obesity	112 (9%)	<10 (<25%)	2,772 (12%)	669 (18%)	2,006 (12%)	582 (16%)
Tobacco Use	105 (8%)	<10 (<25%)	1,939 (9%)	454 (12%)	1,556 (10%)	430 (12%)
Opioid Abuse	< 10 (<1%)	0	36 (<1%)	< 10 (<1%)	37 (<1%)	12 (<1%)
Opioid Dependence	< 10 (<1%)	0	67 (<1%)	20 (1%)	88 (1%)	32 (1%)

Our primary outcome was serum testosterone five years after ADT cessation. This was compared across treatment modalities. Our index event was defined as the first prescription of ADT. When analyzing testosterone (T) levels following ADT cessation, short- and long-term time frames (six and 18 months) were expanded by two months on either end to be more inclusive. Practically, this means that short-term ADT was defined as men who were on ADT between four and eight months, and long-term ADT was defined as men who were on ADT between 16 and 20 months. Androgen deprivation therapy use was determined from a combination of prescription data and chart information from TriNetX, necessitating a less strict cutoff for timing.

Our inclusion criteria were age >40 years of age, clinically localized prostate cancer diagnosis, and eugonadal mean baseline T level (>300 ng/dL) before starting ADT as measured within a year before the initiation of therapy [10]. Men were only included if they had a follow-up testosterone level done five years after stopping ADT. Exclusion criteria included any history of surgical castration, prior history of ADT, and men who received any of the following agents: abiraterone, docetaxel, enzalutamide, darolutamide, apalutamide, and radium chloride. Men were excluded if they received ADT for longer than 20 months.

Statistical analysis

After data collection, we calculated the percentage of patients with eugonadal T levels (>300 ng/dL) at five years to determine the percentage of patients who recovered baseline T levels after ADT therapy. The T recovery rates and mean T values were compared across treatment modalities using the chi-squared test and two-sample t-test, respectively, with statistical significance assessed at p<0.05.

## Results

We identified two cohorts of men who received temporary ADT: 28,583 men received LHRH agonist or antagonist therapy, and 20,188 men received combined ADT. The average age of all groups was over 70 years old, and the majority of men were Caucasian. Table 1 above shows the full demographic information of the men included. We began our analysis by identifying the number of men with mean baseline T levels recorded and whether they were eugonadal prior to initiating therapy. Only 10% of men who received short-term LHRH agonist or antagonist therapies and 23% of men who received long-term LHRH therapies had recorded baseline T levels. Similarly, only 23% of men who received short-term combined ADT had recorded baseline T levels, compared to 41% of men who received long-term combined ADT. We analyzed mean baseline T levels per treatment group, as individual T levels were not available. Eugonadal T levels were defined as a T level >300 ng/dL. Both the LHRH and combined ADT groups had mean baseline testosterone values within 1 standard deviation of testosterone deficiency (Table 2). There was a clear and significant reduction in cohort size when inquiries were made to include patients with reported baseline T levels prior to initiation of therapies. Only men with reported baseline T levels from the initially defined cohorts were included in further analysis, indicating why baseline T levels amongst cohorts may have been reported as less than <300 ng/dL.

**Table 2:** Mean baseline testosterone levels per ADT cohort and duration of therapy ADT: Androgen deprivation therapy, LHRH: Luteinizing hormone-releasing hormone

Patient cohorts	Percentage of men with reported baseline testosterone	Mean baseline testosterone
LHRH Agonists +Antagonists 6 months	10% (N=2,469)	367 + 181 ng/dL
LHRH Agonists +Antagonists 18 months	23% (N=895)	182 ±220 ng/dL
Combined ADT 6 months	23% (N=3814)	364 ± 202 ng/dL
Combined ADT 18 months	41% (N=1477)	186 ± 231 ng/dL

At the five-year follow-up after ADT cessation, the majority of patients with data available failed to recover to mean baseline and eugonadal T levels (Tables 3 and 4). Only 11.7% of men receiving short-term LHRH antagonist or agonist therapy had a follow-up of their T level at five years, while 97.3% of men receiving short-term combined ADT had a follow-up of their T level at five years. Regarding men who received long-term hormone therapies, only 6.8% of LHRH agonist or antagonist recipients and 28.1% of combined ADT recipients had a follow-up T level measured at five years. The large discrepancy in the number of men with follow-up T levels is seen because only men with mean baseline levels prior to initiating therapy were included in this follow-up for comparison. A total of 46.7% of men receiving short-term LHRH agonists or antagonists and 40.6% of patients receiving short-term combined ADT recovered to mean baseline T levels at five years (Table 3). However, only men in the short-term LHRH agonist or antagonist group recovered to eugonadal levels at the five-year follow-up. Amongst the long-term ADT groups, 50% of men who received LHRH agonist or antagonist therapy and 10.7% of patients in the combined ADT group recovered to mean baseline T levels at five years (p<0.0001, Table 4), but neither group recovered to eugonadal T levels. There were no differences in mean testosterone levels at five years between treatment modalities.

**Table 3:** Testosterone recovery in patients after short-term ADT (six months) Mean baseline testosterone levels following short-term ADT at the five-year follow-up. Represented here are the percentage of men with prior baseline T levels recorded and whose subsequent follow-up T levels were measured. The T recovery includes patients who recovered to eugonadal or baseline levels. T: Testosterone, ADT: Androgen deprivation therapy, LHRH: Luteinizing hormone-releasing hormone

ADT modality	Percentage of men with reported five-year follow-up T of those with baseline T recorded	T recovery at the five-year follow-up	P-value	Mean testosterone at 5 years	P-value
LHRH Agonist + Antagonist	11.7%	46.7%	Ref	307.384 + 208.301 ng/dL	Ref
Combined ADT	97.3%	40.6%	0.0556	285.159 + 181.608 ng/dL	0.1900

**Table 4:** Testosterone recovery in patients after long-term ADT (18 months) Mean baseline testosterone levels following long-term ADT at the five-year follow-up. The percentage of men with prior baseline T levels recorded and subsequent follow-up T levels measured are also represented here. The T recovery includes patients who recovered to eugonadal or baseline levels. T: Testosterone, ADT: Androgen deprivation therapy, LHRH: Luteinizing hormone-releasing hormone

ADT modality	Percentage of men with reported five-year follow-up T of those with baseline T recorded	T recovery five-year follow-up	P-value	Mean testosterone at 5 years	P-value
LHRH Agonist + Antagonist	6.8%	50.0%	Ref	255.187 + 161.803	Ref
Combined ADT	28.1%	10.7%	<0.0001	256.757 + 186.096 ng/dL	0.9333

## Discussion

Previous studies have suggested that temporary ADT can cause persistently low T levels, long after stopping ADT [2,8,11-13]. Understanding factors that can cause persistent suppression of T is important so that these at-risk men can be identified and treated. We used a large, national database to identify men with prostate cancer who received temporary ADT to identify if specific types of ADT or durations of ADT impacted the ability of men to recover their T levels. 

We found that the majority of patients undergoing both short-term and long-term ADT failed to recover their T to eugonadal levels five years after treatment cessation. Men who received LHRH agonist or antagonist therapy were more likely to recover their T levels closer to baseline when compared to combined ADT. Additionally, men who received short-term LHRH agonist/antagonist therapy were more likely to regain a higher T level than men on long-term LHRH agonist/antagonist therapy after ADT cessation. Combined ADT recipients did not recover eugonadal baseline T levels at the five-year follow-up. However, short-term combined ADT recipients had higher mean T levels when compared to long-term combined ADT recipients five years after ADT cessation. Therefore, our study supports prior work demonstrating that the duration of ADT affects long-term T levels regardless of medication choice. 

Notably, in certain patient cohorts, such as in short- and long-term combined ADT groups, men had hypogonadal baseline T levels. These men recovered to baseline T levels five years after treatment cessation but did not achieve eugonadal T levels. The T recovery for these men appeared higher when compared to other groups, which may be explained by the reduction in the percentage of men with reported five-year follow-ups when compared to the number of men with baseline T levels.

There is a scarcity of research explaining the variation of T recovery profiles in men undergoing different types of ADT. A previous study of men on flutamide and leuprolide for nine months showed a median time to achieve a eugonadal T level was approximately 10 months after cessation, with 30% of men failing to achieve this level two years after the cessation of ADT [14]. Nascimento et al. evaluated 307 men who underwent ADT for prostate cancer with various agents, including gonadotropin hormone-releasing hormone (GnRH) agonists and antagonists for a mean duration of 17 months, and demonstrated that 24 months after cessation of ADT treatment, 8% of men remained at castrate level; additionally, 76% of men returned to a total T level > 300 ng/dl and 51% returned to baseline testosterone level [2]. In their study, lower baseline T levels and ADT duration > 6 months were associated with a lower likelihood of recovery to normal T at 24 months [2]. Thus, though a considerable number of men seemingly achieve T recovery following ADT suppression, it is difficult to specify which particular mechanism might be most contributory to persistent ADT suppression without longer-term studies with robust sample sizes. To answer this question, Spiegel et al. examined localized prostate cancer patients treated with ADT and radiation therapy and developed a nomogram to estimate T recovery [13]. Factors such as “baseline T levels, duration of ADT, body mass index, age, and race” were predictive in estimating the likelihood of post-ADT, testosterone recovery [13].

The strengths of our study include the use of a large multi-institutional database with a diverse subset of patients across over 40 healthcare organizations. We were able to filter our patient cohorts to include specific inclusion criteria such as ADT modality and patient age. The database provided comprehensive demographic data as well as relevant labs and diagnoses. We are also amongst the few studies in the literature to characterize T recovery following varying types of ADT medication and duration. Additionally, due to a lack of specific information regarding the details of prostate cancer treatments, we were unable to account for radiation modalities and the variation in effects on testosterone production. One main limitation is the lack of men without a baseline T level and the limited number of men with a T level drawn at the five-year follow-up. However, this has been a challenge for prior studies as well and could be due to uncaptured mortality. In a study by Nascimientao et al., 81% of patients in their study of early T recovery in patients treated with ADT did not have baseline T levels reported [2]. It is critical to identify baseline T levels for managing expectations in patients undergoing ADT therapy. The provider should discuss the risk of incomplete T recovery in this patient population, especially for those with low baseline T. Another important limitation was that we were unable to determine if these men were symptomatic from low T due to limitations inherent in the TriNetX database.

Future studies on T recovery profiles following ADT cessation should include further analysis of T levels amongst varying ADT treatment regimen groups with longer-term follow-up. This will be especially important as new therapeutic options emerge for ADT therapy. We encourage providers to evaluate baseline T levels in all patients before initiating ADT therapy and to ensure monitoring of post-treatment T levels on a long-term basis to prevent the sequela of undiagnosed persistent T deficiency.

## Conclusions

Regardless of the ADT medication type, testosterone recovery to baseline at five years following temporary therapy for localized prostate cancer appears incomplete. Men receiving ADT should have baseline and post-treatment testosterone levels monitored to detect and prevent the sequela of clinically significant testosterone deficiency.
